# The influence of aerobic exercise on model-based decision making in women with posttraumatic stress disorder

**DOI:** 10.1016/j.xjmad.2023.100015

**Published:** 2023-07-27

**Authors:** Kevin M. Crombie, Ameera Azar, Chloe Botsford, Mickela Heilicher, Jaryd Hiser, Nicole Moughrabi, Tijana Sagorac Gruichich, Chloe M. Schomaker, Josh M. Cisler

**Affiliations:** a The University of Texas at Austin, Department of Psychiatry and Behavioral Sciences, 1601 Trinity Street, Building B, Austin, TX 78712, United States; b The University of Alabama, Department of Kinesiology, 1003 Wade Hall, Tuscaloosa, AL 35487, United States; c University of Wisconsin – Madison, Department of Psychiatry, 6001 Research Park Boulevard, Madison, WI 53719, United States; d The Ohio State University, Department of Psychiatry and Behavioral Health, 1670 Upham Drive, Suite 130, Columbus, OH 43210, United States; e Institute for Early Life Adversity Research, The University of Texas at Austin Dell Medical School, 1601 Trinity Street, Building B, Austin, TX 78712, United States

**Keywords:** Reinforcement learning, Habit, Two-stage Markov task, Cognition, PTSD

## Abstract

Individuals with PTSD often exhibit deficits in executive functioning. An unexplored aspect of neurocognitive functions associated with PTSD is the type of learning system engaged in during decision-making. A model-free (MF) system is habitual in nature and involves trial-and-error learning that is often updated based on the most recent experience (e.g., repeat action if rewarded). A model-based (MB) system is goal-directed in nature and involves the development of an abstract representation of the environment to facilitate decisions (e.g., choose sequence of actions according to current contextual state and predicted outcomes). The existing neurocognitive literature on PTSD suggests the hypothesis of greater reliance on MF vs MB learning strategies when navigating their environment. While MF systems may be more cognitively efficient, they do not afford flexibility when making prospective predictions about likely outcomes of different decision-tree branches. Emerging research suggests that an acute bout of aerobic exercise improves certain aspects of neurocognition, and thereby could promote the utilization of MB over MF systems during decision making, although prior research has not yet tested this hypothesis. Accordingly, the current study administered a lab-based two-stage Markov decision-making task capable of discriminating MF vs MB decision making, in order to determine if moderate-intensity aerobic exercise (either shortly after or 30-minutes after the exercise bout has ended) promotes greater engagement in MB behavioral strategies compared to light-intensity aerobic exercise in adult women with and without PTSD (N = 61). Results revealed that control women generally displayed higher levels of MB behavior that was further increased following immediate exercise, particularly moderate-intensity exercise. By contrast, the PTSD group generally displayed lower levels of MB behavior, and exhibited greater MB behavior when completing the task following moderate-intensity aerobic exercise compared to light-intensity aerobic exercise regardless of whether there was a short or long delay between exercise and the task. Additionally, women with PTSD demonstrated less impairment in MB decision-making compared to controls following moderate-intensity aerobic exercise. These results suggest that an acute bout of moderate-intensity aerobic exercise boosts MB behavior in women with PTSD, and suggests that aerobic exercise may play an important role in enhancing cognitive outcomes for PTSD.

## 1 Introduction

Humans make decisions every day. Many decision-making contexts require navigating multi-step decisions towards the ultimate obtainment of meaningful outcomes (e.g., selecting which roads to take on evening commute to avoid traffic; make decisions to maximize likelihood of a monetary gain) [1]. Sequential decision making is thought to arise from a combination of 1) simple trial-and-error learning (termed model-free learning) where action values are often updated based on the most recent experience, and 2) more complex learning in which individuals form an abstract representation of the environment which allows them to make prospective predictions about likely outcomes based on different choices (termed model-based learning) [2], [3]. To illustrate these different approaches using the above example, consider an individual who is about to make their evening commute on a Friday afternoon. An individual relying on a model-free strategy would simply take the route that they took yesterday (through downtown) because there was less than usual traffic and they arrived home at a reasonable time. In contrast, an individual utilizing more of a model-based strategy would take into consideration that it is rush hour on a Friday afternoon with thousands of commuters trying to get downtown for a concert, and instead will take an alternative route (through county highways) that may be longer in distance, but will actually result in them arriving at their destination sooner than the downtown route that was the best option yesterday.

Model-based learning is often thought to be desirable and advantageous in many contexts as an individual utilizing a model-based approach has developed a more informed cognitive map, but it is also more cognitively demanding as it requires the formation of cognitive representations of the environment to assist with goal-directed choices. In other words, it requires adequate utilization of processes involved in higher-level executive functioning (e.g., working memory, cognitive control, use of abstract rules or instruction) [4], [5], [6]. As such, individuals that exhibit deficits in executive functioning may be more prone to utilize model-free as opposed to model-based learning strategies when navigating their environment. Emerging evidence suggests that individuals with posttraumatic stress disorder (PTSD) may be one such population that is characterized by greater utilization of habit-based (model-free) rather than prospective (model-based) decision-making systems. For instance, prior research suggests that neuropsychological functioning (including executive functioning) is often impaired in PTSD [7], [8], [9], [10], and trauma exposure severity (particularly interpersonal violence trauma severity) is associated with decreased model-based learning for reward [11]. In fact, individuals with PTSD often avoid seeking rewarding or pleasurable experiences because of incorrectly formed models about the world constantly being a dangerous place [12].

As such, we hypothesize that greater engagement in model-based learning may provide individuals with PTSD more flexibility to make better informed prospective predictions about likely outcomes of their decisions. Although prior research has not yet tested this possibility, emerging research suggests that an acute bout of aerobic exercise could promote the utilization of model-based over model-free systems during decision making. For instance, aerobic exercise acutely increases performance on several cognitive tasks, including executive functioning and memory tasks (e.g., verbal fluency, decision-making, visual short-term memory, free recall [13], [14]). Additionally, aerobic exercise engages several neurotransmitters/neurotransmitter modulators (e.g., endocannabinoids), growth factors (e.g., brain derived neurotrophic factor), and brain regions (e.g., hippocampus, prefrontal cortex) that are relevant for higher-level cognitive processes, including decision making [15], [16], [17], [18], [19], [20]. Relatedly, enhancing or diminishing activity within the frontoparietal network (a network involved in executive functioning and engaged during model-based learning) was accompanied by enhanced or diminished model-based learning, respectively [21], [22], [23], [24], [25]. Based on these converging pieces of evidence, it follows that aerobic exercise may also increase model-based learning. Thus, an important first step is to determine whether an acute bout of aerobic exercise increases engagement of model-based behavioral control during a decision-making task.

Moreover, it is also critical to determine if the potential effect of aerobic exercise differs as a function of when the task is performed in relation to exercise. In other words, if we want to know if aerobic exercise impacts model-based learning, a related relevant question is to ask for how long does the impact of exercise on model-based learning persist? Previous meta-analytic evidence suggests that cognitive tests administered shortly after (albeit not immediately after) the exercise bout has ended generally results in a greater effect on cognitive performance compared to when the task is performed following a delay of 20-minutes or more [13]. Finally, given that adults with PTSD often exhibit impairments in executive functioning, it is also important to determine whether there are differential effects for women with and without PTSD. Accordingly, the current study had women with and without PTSD engage in acute bouts of moderate-intensity aerobic exercise and a light-intensity control condition (separated by one week) before completing a lab-based two-stage Markov decision-making task (either immediately after exercise/control or 30 mins later) capable of discriminating model-free from model-based control in order to determine if moderate-intensity aerobic exercise promotes greater engagement in model-based behavioral strategies compared to a light-intensity aerobic exercise, and whether the effect of exercise differs as a function of the timing of the task administration and/or PTSD diagnosis.

## 2 Materials and methods

All procedures were approved by the Health Sciences Institutional Review Board at the University of Wisconsin—Madison and the Institutional Review Board at the University of Texas at Austin. The work described in this manuscript has been carried out in accordance with the Code of Ethics of the World Medical Association (Declaration of Helsinki) for experiments involving humans.

### 2.1 Study participants

Participants were recruited from general community-based advertising including newspaper and online advertisements (e.g., Craigslist, Facebook, campus event pages), and flyers posted at approved locations on the University of Wisconsin-Madison and The University of Texas at Austin campuses and at health clinics throughout the greater Madison, WI and Austin, TX areas. Interested individuals underwent a phone screen to assess potential eligibility. Two groups (control, PTSD) of participants were recruited for this study. Inclusion criteria for the control group consisted of: 1) females, 2) 21–50 years of age, and 3) no prior history of interpersonal assaultive violence exposure (i.e., directly witnessed or experienced exposure to physical or sexual assault). Inclusion criteria for the PTSD group consisted of: 1) females, 2) 21–50 years of age, and 3) a current diagnosis of PTSD where the index event involved interpersonal assaultive violence exposure [26], [27], [28], [29].

Exclusion criteria for both groups included: 1) being pregnant or planning to become pregnant; 2) having a history of physical discomfort, difficulty, light headedness or fainting during blood draws or physical activity; 3) having a history of chest pain during physical activity; 4) having a bone, joint, or other medical condition that may be worsened by physical activity; 5) having asthma (unless controlled with medication, inhaler, or lifestyle change); 6) responding ‘yes’ to any of the seven questions on the Physical Activity Readiness Questionnaire (unless participant provided a doctor’s note indicating that it is safe for them to engage in moderate-intensity aerobic exercise); 7) percutaneous coronary intervention or acute myocardial infarction in the last 6 weeks; 8) unstable arrhythmias/implanted cardiac defibrillator shocks in the last 3 months; 9) indication of suicidality; 10) presence of major medical disorder (e.g., cancer, HIV); 11) having a current or past diagnosis of any psychotic disorder (e.g., schizophrenia); 12) cognitive/intellectual disabilities; 13) developmental disorders; 14) active substance use disorders; 15) glaucoma, 16) vulnerable populations (e.g., appearing to lack consent capacity); and 17) any other condition that the investigator believed might put the participant at risk.

Psychiatric medications were not exclusionary if the medication was stable for at least 4 weeks. Acute sedatives / pain killers (e.g., benzodiazepines, opioids) and prescription stimulants (e.g., methylphenidate, amphetamines) were not permitted for 6 h prior to study visits 1 and 2 (experimental sessions). To facilitate generalization to real-world populations of women with assault-related PTSD [30], [31], current comorbid major depressive disorder or anxiety disorders were not exclusionary if the PTSD diagnosis was primary.

### 2.2 Clinical assessment

During the remote intake assessment visit (study visit 0), participants were administered a series of structured clinical interviews, including the Clinician Administered PTSD Scale (CAPS; [32], [33]), National Women Survey Trauma Assessment (NSA; [28]), Diagnostic Interview for Anxiety, Mood, and Compulsive Related Neuropsychiatric Disorders (DIAMOND; [34]), and the Columbia-Suicide Severity Rating Scale (CSSRS; [35]). Additionally, participants completed a neurocognitive task (vocabulary and matrix reasoning subscales of Wechsler Abbreviated Scale of Intelligence [WASI-II; [36]) to quantify IQ, and provided demographic information such as age, race, ethnicity, income, etc. Eligible participants were then sent a secured REDCap link to complete baseline questionnaires, including: Beck Depression Inventory-II (BDI-II; [37]), Beck Anxiety Inventory (BAI; [38]), Posttraumatic Stress Disorder Checklist-5 (PCL-5; [39]), Childhood Trauma Questionnaire (CTQ; [40]), Difficulty in Emotion Regulation Scale (DERS; [41]), Pittsburgh Sleep Quality Index (PSQI; [42]), Mood and Anxiety Symptoms Questionnaire (MASQ D-30; [43]), Perceived Stress Scale (PSS; [44]), and a menstrual cycle log.

### 2.3 Randomization

The current study involved within- and between-subjects randomization procedures. For the within-subject manipulation, participants were randomized to complete either light- or moderate-intensity aerobic exercise at study visit 1 (with remaining condition completed at study visit 2). For the between-subject manipulation, participants were randomized to complete the two-stage Markov decision making task either shortly after (short-delay condition; time to complete questionnaires and blood draw) or 30-minutes following (long-delay condition) the exercise manipulation (i.e., following light- or moderate-intensity aerobic exercise). The randomization factors were crossed, resulting in 4 experimental groups (see Fig. S1). In other words, a quarter of participants completed the moderate-intensity/short-delay condition at study visit 1 (and the light-intensity/short-delay condition at study visit 2), a quarter of participants completed the moderate-intensity/long-delay condition at study visit 1 (and the light-intensity/long-delay condition at study visit 2), a quarter of participants completed the light-intensity/short-delay condition at study visit 1 (and the moderate-intensity/short-delay condition at study visit 2), and a quarter of participants completed the light-intensity/long-delay condition at study visit 1 (and the moderate-intensity/long-delay condition at study visit 2). Randomization was carried out using blocked and stratified randomization procedures (using custom code in MATLAB), in which randomization was stratified based on age (>35 or <= 35) and menstrual cycle (regular cycle vs irregular cycle, birth control, or menopause). Participants were not provided information as to which condition (moderate- or light-intensity) they were to complete/completed at either visit (information was provided at debriefing session).

### 2.4 Study overview

This study involved three main stages: an intake assessment (study visit 0), and two experimental sessions during which participants completed the two-stage Markov decision making task following either the light- or moderate-intensity aerobic exercise conditions (study visits 1 and 2). Participants completed these stages across three separate days. Study visits 1 and 2 took place no more than 30 days after study visit 0. Study visits 1 and 2 occurred at approximately the same time of day for each participant (difference in start time: M = 0.05 h, SD = 0.46 h; time of day [military time]: M = 10.91, SD = 2.95; 82% of visits occurred before 12PM CST). Study visits 1 and 2 occurred approximately 7 days apart (87% of visits occurred exactly seven days apart; M = 6.90, SD = 0.66), although up to 14 days apart was allowed to accommodate participant availability (note: 5 days was shortest interval [n = 4] and 9 days was longest interval [n = 1] in between visits).

During study visit 1, participants were provided with task instructions, completed a quiz to verify they understood the task, and completed practice trials (see below for further details). Participants then completed the State Trait Anxiety Inventory – State Version (STAI; [45]) and Positive and Negative Affect Schedule (PANAS; [46]), and had their blood drawn before being administered either the light- or moderate-intensity aerobic exercise condition (see below for additional information; note: blood draws were not attempted on 14 participants due to global supply issues). Following exercise, participants again had their blood drawn, completed the STAI and PANAS, and then completed the two-stage Markov decision making task (either immediately after completing questionnaires or 30-minutes after the experimental condition ended; see randomization procedures). Study visit 2 was identical to study visit 1, as the only difference was that participants completed the remaining experimental condition (light- or moderate-intensity aerobic exercise) that was not completed at study visit 1. Specific details regarding experimental procedures can be found below. Participants were compensated for $50 for completing the intake visit, and an additional $100 after completing the two in-person study visits.

### 2.5 Two stage Markov behavioral decision-making task

Our two stage Markov decision-making task was adapted from a recent implementation of this task using ‘magic carpet’ story-based instructions (detailed in [47]). Prior tasks using incomplete story-based instructions and task descriptions have found that humans generally use a mix of model-free and model-based learning strategies. By contrast, this prior study demonstrated that when a cover story is used that describes all of the task elements and transitions, humans predominantly rely on model-based learning strategies [47], and the apparent use of model-free strategies is most likely due to incomplete understanding of the task and/or using an internal model that does not correspond to the correct model underlying the task. As such, using the ‘magic carpet’ task instructions ensured correct understanding of the task thereby promoting model-based learning. During the instructions phase (completed at start of each visit), participants completed a tutorial that conveyed the task story and implicitly introduced key concepts (e.g., probabilistic rewards, transition probabilities; see Fig. S2). At the end of the tutorial, participants completed a practice quiz (consisting of seven multiple choice questions) to ensure they understood the task, followed by 25 practice trials (using different symbols and colors for first stage and second stage choices) during which participants explicitly knew whether transitions from the first to second stage were common or rare (which is in contrast to actual task; see Supplemental Material and Methods for complete overview of tutorial instructions and screen-by-screen presentations of task [Fig. S2]).

**Fig. 1:**
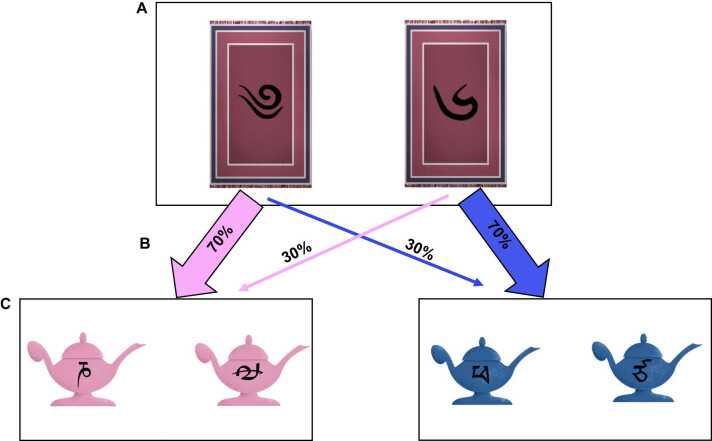
Schematic of two-stage Markov decision making task. On each trial, participants must first choose one of two ‘magic carpets’, which were labeled with two different symbols (Tibetan characters) that signified ‘Blue Mountain’ or ‘Pink Mountain’ in the local language (i.e., stage 1 choice; see panel A). Each carpet was associated with a greater likelihood of taking the participant to either the ‘Blue Mountain’ or ‘Pink Mountain’ (i.e., on each trial participants either experienced a common or a rare transition to stage 2; see panel B). Upon arriving on a mountain, participants were then required to choose between one of two lamps, which were labeled with two different symbols (Tibetan characters) that signified the name of the genie inside the lamp (i.e., stage 2 choice; see Panel C). Participants then received either a reward (i.e., image of a genie giving them a golden coin) or no reward (i.e., image of a zero) according to a slowly drifting probability (between 0.25 and 0.75). Participants completed 250 trials in five blocks separated by 30-second rest periods.

Participants were tasked with trying to accumulate gold coins from genies who lived inside magic lamps on Pink and Blue Mountains. First, participants chose between one of two magic carpets which were labeled with two different symbols (Tibetan characters) that signified “Blue Mountain” and “Pink Mountain” in the local language (first-stage choice). Each carpet traveled more frequently to one of the mountains (70%) than the other mountain (30%). For example, one of the magic carpets had a 70% probability of landing on the Blue Mountain (common transition) and a 30% probability of landing on the Pink Mountain (rare transition). The other magic carpet had the opposite probabilities (i.e., 70% chance of landing on Pink Mountain and 30% chance of landing on Blue Mountain). Upon arriving on a mountain, participants chose between one of two lamps, which were labeled with two different symbols (Tibetan characters) that signified the name of the genie inside the lamp (second stage choice). Participants then received either a reward (i.e., image of a genie giving them a golden coin) or no reward (i.e., image of a zero) according to a slowly drifting probability (between 0.25 and 0.75). Participants completed 250 trials in five blocks separated by 30-second rest periods (see Fig. 1).

Two stage Markov decision making tasks (including the task administered in this study) aim to distinguish between model-based and model-free behavioral learning strategies [2], [3]. In general, an individual utilizing a model-based behavioral strategy forms a cognitive map/model of transition types (rare or common) and outcomes (rewarded or unrewarded) in the task to aid in their selection on subsequent trials (e.g., change selection at first stage on next trial if rewarded following a rare transition to second stage on previous trial). In contrast, an individual utilizing a model-free behavioral strategy would not form a cognitive map/model of transition types and outcomes in the task, and instead may simply base their selection on subsequent trials on whether the previous trial was rewarded (i.e., repeat selection on next trial if rewarded or change selection on next trial if unrewarded, regardless of whether transition to second stage was rare or common), or they may base their decision on all past trial action outcomes (temporally discounted) in calculating whether to repeat or switch their selection on the next trial. For example, consider a rewarded trial in which an individual selects a magic carpet that is intended to travel to the Blue Mountain, but instead takes them to the Pink Mountain (rare transition). An individual implementing a model-based behavioral strategy would consider the transition type and probabilities and therefore would be more likely to switch their selection at the first stage of the next trial (select opposite carpet), in an attempt to get back to the Pink Mountain (for which they can expect to receive a reward upon repeating their choice at the second stage). An individual implementing a model-free behavioral strategy would likely simply repeat the same choice (select carpet more likely to go to Blue Mountain) on the next trial since they were rewarded on the previous trial. In other words, model-free behavior results in a main effect of reward, whereas model-based behavior results in a reward x transition interaction. As such, we operationalized the use of model-based learning (vs model-free learning) through the magnitude of the beta-coefficient for the reward x transition interaction from logistic regression models (described below). See Fig. 3A for prototypical examples of model-free vs model-based responding on this task.

### 2.6 Aerobic exercise procedures

The moderate-intensity aerobic exercise condition consisted of a 5-minute warm-up of very-light to light-intensity activity (40–50% age-adjusted maximum heart rate; MHR) on stationary cycle ergometer (Lode Corival cpet, Lode BV, Gronigen, The Netherlands) followed by cycling at a moderate-intensity (70–75% MHR) for 30-minutes. The same cycle ergometer was used at both research sites. Participants were instructed to maintain a pedal rate of 60–70 revolutions per minute, as pedal resistance (watts) was adjusted until the participant reached their target heart rate. At the end of the 30-minute session, participants cooled down for 5 min at a light-intensity. The light-intensity condition consisted of very-light to light-intensity activity (40–50% MHR) on a stationary cycle ergometer for the full duration of 40-minutes. Heart rate (assessed via Polar heart rate chest strap) and cycling parameters (RPM, watts) were continuously monitored throughout the session, and heart rate and ratings of perceived exertion (RPE) were recorded every five minutes throughout the respective sessions. Watts were adjusted (if necessary) throughout the exercise session in order to keep participants in their MHR range.

### 2.7 Baseline characteristics and exercise session analyses

Descriptive statistics (means and standard deviations) were calculated for all baseline outcomes and exercise session parameters. Potential differences between groups (for all baseline assessment measures) were analyzed with independent samples t-test (for parametric data) and Wilcoxon rank sum tests (for non-parametric data). Potential differences between conditions for the exercise session parameters (i.e., percent of maximum heart rate; ratings of perceived exertion, RPM, watts) were analyzed with paired samples t-tests.

### 2.8 Logistic regression analyses of stay/switch behavior

We analyzed trial-by-trial stay vs switch behavior using logistic regression mixed models. The decision to use a logistic regression model over a computational model (e.g., hybrid model) was based on prior work (using this task) that revealed that a logistic regression model was significantly better at explaining participant’s first stage choices compared to a hybrid and model-based computational models [47]. The primary model was performed (in MATLAB using fitglme specifying a binomial distribution and logit function) to determine the likelihood of participants staying or switching their behavior (i.e., choosing the same or a different carpet) at the first stage as a function of reward outcome during the previous trial (rewarded vs no reward), transition type during the previous trial (common vs rare transition), experimental condition (light- vs moderate-intensity), delay condition (short-delay vs long-delay), group (control vs PTSD), visit (visit 1 vs visit 2), site (UW and UT), and age (mean-centered; included as fully interactive covariate due to group difference in mean age) with an additional covariate for IQ, with random effects for experimental condition, visit, and experimental condition x visit interaction by subject. Due to large degrees of freedom in the logistic regression analyses and number of predictors and interaction terms, only effects from the omnibus model with a *p* value less than .001 were interpreted. Given the purpose of the study, we focused on interpreting effects related to exercise and PTSD. The data that support the findings of this study are available from the corresponding author upon reasonable request.

## 3 Results

### 3.1 Participant enrollment, retention, and data analysis

See Supplementary Fig. S3 for a complete overview of participant enrollment flow and retention. 142 individuals expressed interest in the study and were assessed for eligibility. 72 individuals were excluded for not meeting inclusion/exclusion criteria (n = 51) or were no longer interested in participating after the initial phone screen. Seventy individuals were enrolled in the study and randomly assigned to receive experimental conditions. Thirty-three control individuals were randomly assigned and 32 of these individuals completed both experimental sessions (one individual dropped out after the intake). Two individuals from the control group were excluded from the analyses due to providing the same response throughout the entire task. Thirty-seven individuals with PTSD were randomly assigned and 31 of these individuals completed at least one experimental session (6 individuals dropped out after intake). Two individuals from the PTSD group dropped out after completing only 1 visit and data from one individual’s session was excluded due to falling asleep during the delay period.

### 3.2 Participant characteristics

See Table 1 for complete overview of participant characteristics for primary variables of interest (e.g., age, race, IQ, depression and PTSD symptom severity). There were no group differences for all baseline assessments between the short-delay and long-delay conditions within the control and PTSD groups. There were no group differences between control and PTSD groups for education, IQ, and race. The PTSD group was on average older, and exhibited greater anxiety and depressive symptom severity (obtained via BDI-II and MASQ-D30), perceived stress (obtained via PSS), and childhood trauma (obtained via CTQ) compared to the control group. Additional secondary baseline variables characterizing the sample can be found in Table S1.

**Table 1:** Group means, standard deviations, and frequencies for baseline characteristics.

	Control Group (n = 30)				PTSD Group (n = 31)				Between Group (CON vs PTSD) Statistics	
Variable	Short Delay	Long Delay	t-statistic	p-value	Short Delay	Long Delay	t-statistic	p-value	t-statistic	p-value
**Age** (yrs)	28.73 ± 7.79	30.40 ± 8.54	-0.56	0.581	36.20 ± 9.17	37.94 ± 9.33	-0.52	0.606	-3.40	0.001
**Education** (yrs)	16.40 ± 2.10	16.53 ± 1.41	-0.20	0.840	16.93 ± 2.87	16.31 ± 3.20	0.57	0.575	-0.23	0.818
**IQ** (FSIQ2)	119.53 ± 10.74	113.37 ± 9.25	1.18	0.241	108.87 ± 11.58	114.81 ± 15.48	-1.20	0.238	0.70	0.485
**Race**			-1.54	0.124			0.00	0.999	-0.26	0.798
# (% White)	12 (80.00)	8 (53.33)			10 (66.67)	10 (62.50)				
# (% Black/African-American)	0 (0.00)	0 (0.00)			0 (0.00)	1 (6.25)				
# (% Asian)	2 (13.33)	3 (20.00)			1 (6.67)	1 (6.25)				
# (% Hispanic/Latino)	0 (0.00)	2 (13.33)			2 (13.33)	3 (18.75))				
# (% Other)	1 (6.67)	2 (13.33)			2 (13.33)	1 (6.25)				
**Depression** (BDI-II)	5.33 ± 5.67	3.60 ± 4.23	0.95	0.353	15.47 ± 9.41	21.31 ± 8.57	-1.81	0.081	-7.28	< 0.001
**Perceived Stress** (PSS)	15.20 ± 5.67	12.53 ± 4.26	1.46	0.156	22.87 ± 6.49	24.00 ± 5.15	-0.54	0.592	-6.90	< 0.001
**Mood and Anxiety Symptoms** (MASQ-D30)										
Anhedonic depression	32.80 ± 8.01	27.53 ± 6.74	1.95	0.061	37.67 ± 9.18	39.69 ± 4.51	-0.79	0.438	-4.49	< 0.001
Anxious arousal	13.67 ± 4.20	12.20 ± 3.21	1.07	0.292	17.33 ± 4.88	20.38 ± 5.14	-1.69	0.102	-5.15	< 0.001
General distress	16.67 ± 5.43	14.67 ± 3.39	1.21	0.237	26.07 ± 7.75	29.38 ± 6.70	-1.27	0.213	-7.37	< 0.001
**Childhood Trauma** (CTQ)										
Emotional abuse	7.80 ± 3.74	7.20 ± 4.07	0.42	0.678	15.20 ± 5.97	13.38 ± 5.77	0.87	0.394	-5.31	< 0.001
Emotional neglect	8.07 ± 3.20	7.07 ± 1.53	1.09	0.283	16.67 ± 5.26	13.88 ± 4.99	1.52	0.140	-7.24	< 0.001
Minimization-denial	9.47 ± 1.55	9.87 ± 1.19	-0.79	0.435	7.47 ± 0.99	8.38 ± 1.78	-1.74	0.093	4.69	< 0.001
Physical abuse	5.47 ± 1.24	5.67 ± 0.90	-0.50	0.618	9.20 ± 5.24	9.25 ± 3.73	-0.03	0.976	-4.38	< 0.001
Physical neglect	5.80 ± 1.78	5.53 ± 1.30	0.47	0.643	10.00 ± 3.63	9.06 ± 4.07	0.68	0.505	-5.12	< 0.001
Sexual abuse	5.00 ± 0.00	5.00 ± 0.00	-	-	9.60 ± 6.37	9.25 ± 5.46	0.16	0.870	-4.16	< 0.001
Total	41.60 ± 6.91	40.33 ± 5.75	0.55	0.590	68.13 ± 18.49	63.19 ± 17.88	0.76	0.455	-7.07	< 0.001
**PTSD Symptom Severity** (CAPS-5)										
Cluster B: Reexperiencing/intrusive	-	-	-	-	7.79 ± 2.97	9.00 ± 2.53	-1.21	0.236	-	-
Cluster C: Avoidance	-	-	-	-	4.14 ± 1.51	4.19 ± 1.68	-0.08	0.940	-	-
Cluster D: Negative alterations in cognition/mood	-	-	-	-	10.93 ± 3.99	13.56 ± 3.27	-1.98	0.057	-	-
Cluster E: Marked alterations in arousal/reactivity	-	-	-	-	7.86 ± 3.24	7.69 ± 2.89	0.15	0.882	-	-
Total PTSD symptom severity	-	-	-	-	30.71 ± 9.16	34.44 ± 6.40	-1.30	0.202	-	-

Note. Values listed as M ± SD unless otherwise noted. Potential differences between groups were analyzed with independent samples t-test except for race (nominal data) which was analyzed with Wilcoxon rank sum tests (and therefore reported test statistics are Z-values). There were no significant group differences between short delay and long delay conditions within both the control and PTSD groups. There were no significant differences between the control and PTSD groups for education, IQ, or race. The PTSD group was older and reported greater depressive symptom severity, perceived stress, mood and anxiety symptoms, and childhood trauma compared to the control group. PTSD = Posttraumatic Stress Disorder; FSIQ-2 = Full Scale-2 IQ (obtained from Wechsler Abbreviated Scale of Intelligence-II [WASI-II]); BDI-II = Beck Depression Inventory-II; PSS = Perceived Stress Scale; MASQ-D30 = Mood and Anxiety Symptoms Questionnaire-30 items; CTQ = Childhood Trauma Questionnaire; CAPS-5 = Clinician Administered PTSD Scale for DSM-V.

### 3.3 Exercise session

See Table 2 for complete summary of exercise session parameters. All control (short- and long-delay) and PTSD (short- and long-delay) groups exhibited significantly greater ratings of perceived exertion, watts, and exercising heart rate (beats per minute and percent of maximum) during the moderate-intensity aerobic exercise condition compared to the light-intensity condition. As intended, based on heart rate measures and ratings of perceived exertion, all groups exercised at a light-intensity during the light-intensity control condition and at a moderate-intensity during the moderate-intensity aerobic exercise condition.

**Table 2:** Group means and standard deviations for exercise session variables.

	Means, Standard Deviations, and Statistics							
Variable	Light-Short Delay	Moderate-Short Delay	t-statistic	p-value	Light-Long Delay	Moderate-Long Delay	t-statistic	p-value
**Ratings of perceived exertion (RPE)**								
Control Group	9.06 ± 1.44	14.06 ± 1.19	-11.28	< 0.001	8.92 ± 1.40	13.83 ± 2.25	-8.14	< 0.001
PTSD Group	9.62 ± 2.00	13.83 ± 1.28	-7.28	< 0.001	10.47 ± 2.19	14.36 ± 1.76	-5.83	< 0.001
**Exercising heart rate (bpm)**								
Control Group	91.43 ± 10.15	139.48 ± 6.69	-25.37	< 0.001	87.00 ± 7.72	137.59 ± 5.22	-24.55	< 0.001
PTSD Group	90.66 ± 11.42	130.99 ± 10.65	-12.81	< 0.001	88.61 ± 8.59	126.81 ± 7.45	-17.48	< 0.001
**Heart rate percentage (%MHR)**								
Control Group	47.76 ± 4.50	72.92 ± 1.99	-25.25	< 0.001	45.89 ± 3.48	72.62 ± 2.12	-23.88	< 0.001
PTSD Group	49.15 ± 5.79	70.99 ± 4.02	-13.31	< 0.001	48.90 ± 4.57	70.31 ± 2.28	-18.80	< 0.001
**Watts**								
Control Group	13.90 ± 10.25	73.36 ± 26.73	-10.56	< 0.001	18.06 ± 11.48	80.74 ± 26.49	-10.41	< 0.001
PTSD Group	13.87 ± 10.12	69.81 ± 23.31	-12.29	< 0.001	11.51 ± 9.17	63.26 ± 21.32	-10.49	< 0.001
**Pedal rate (RPM)**								
Control Group	66.26 ± 2.72	65.37 ± 2.62	1.84	0.087	67.18 ± 2.18	65.90 ± 2.52	2.40	0.031
PTSD Group	65.22 ± 3.85	63.74 ± 2.32	1.43	0.176	66.14 ± 2.47	64.40 ± 3.37	1.52	0.151

Note. Bpm = beats per minute; MHR = maximum heart rate; RPM = revolutions per minute. Ratings of perceived exertion, exercising heart-rate (bpm and %MHR) and watts were significantly greater during the moderate-intensity aerobic exercise condition vs the light-intensity condition for the control and PTSD groups. All groups maintained a cycle rate in the instructed 60–70 RPM range.

### 3.4 Logistic regression analyses of stay/switch behavior

As expected, there was a reward x transition x visit interaction (*t*(29438) = 2.00, *p* = .044) indicating that participants experienced an increase in model-based behavior from visit 1 to visit 2. Additionally, there was a significant reward x transition x experimental condition x delay condition x group interaction (*t*(29438) = 4.07, *p* < .001). We broke this interaction down by examining the simple effects separately within the PTSD and control groups, and then by examining differences between the PTSD and control groups.

**Fig. 2:**
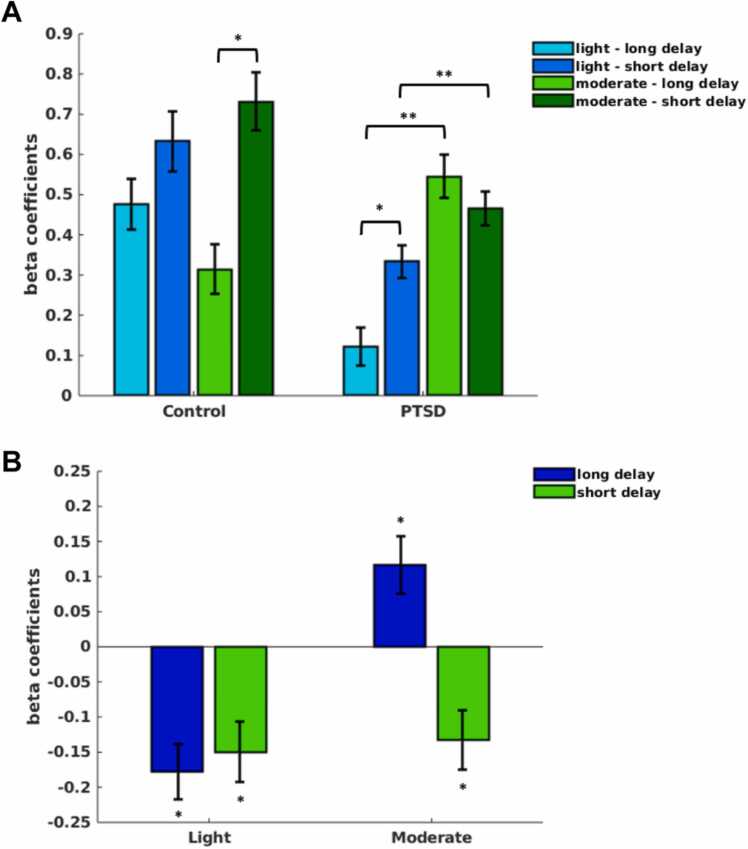
Figure depicting results from logistic regression analyses of stay/switch behavior during two-stage Markov decision-making task. Participants from the PTSD group exhibited greater model-based behavior when completing the task following moderate-intensity aerobic exercise compared to light-intensity aerobic exercise regardless of whether there was a short delay or long delay between exercise and the task (depicted as **; see panel A). Additionally, both the PTSD and control groups exhibited greater model-based behavior following the light-intensity short delay condition compared to the light-intensity long delay condition, and the control group exhibited greater model-based behavior following the moderate-intensity short delay condition compared to the moderate-intensity long delay condition (depicted as *; see panel A). Additionally, the control group exhibited greater model-based behavior during the light-intensity short delay and long delay conditions and the moderate-intensity short delay condition (but not the moderate-intensity long delay condition) compared to the PTSD group (depicted as *; see panel B). Panel A beta-coefficients = reward x transition; Panel B beta-coefficients = reward x transition x group (control vs PTSD).

**Fig. 3:**
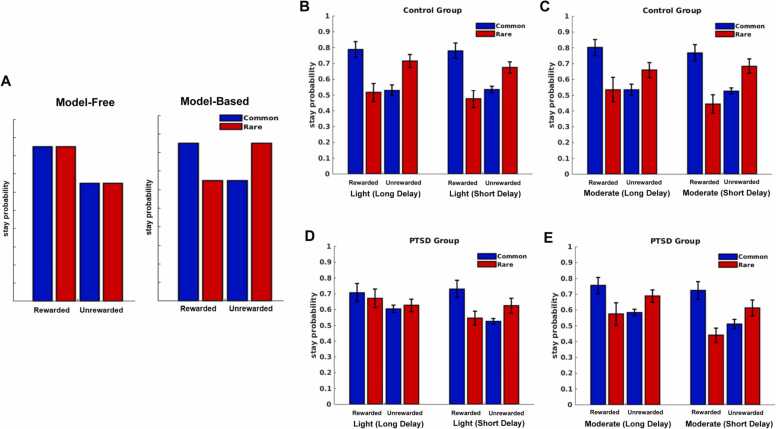
Figure depicting stay probabilities for each group (control and PTSD) across experimental manipulations (light-intensity vs moderate-intensity aerobic exercise and short delay vs long delay). Panel A depicts prototypical model-free and model-based behavior. Prototypical model-free behavior depicts a main effect of reward, where an individual is more likely to repeat their behavior (stay) on the next trial if they are rewarded (regardless of common or rare transition). Prototypical model-based behavior depicts a reward x transition interaction where an individual is more likely to repeat their behavior (stay) on the next trial if they are rewarded following a common transition or if they are unrewarded following a rare transition, and they are more likely to change their behavior (switch) on the next trial if they are rewarded following a rare transition or unrewarded following a common transition (see panel A). Panels B and C depicts stay probabilities for the control group during the light-intensity long delay and short delay conditions (see panel B) and during the moderate-intensity long delay and short delay conditions (see panel C). Panels D and E depicts stay probabilities for the PTSD group during the light-intensity long delay and short delay conditions (see panel D) and during the moderate-intensity long delay and short delay conditions (see panel E). Results indicated that the PTSD group exhibited greater model-based behavior when completing the task following moderate-intensity aerobic exercise compared to light-intensity aerobic exercise regardless of whether there was a short delay (Cohen’s *d* for stay probability on rare rewarded trials = −0.62; Cohen’s *d* for stay probability on rare unrewarded trials = 0.06) or long delay (Cohen’s *d* for stay probability on rare rewarded trials = −0.39; Cohen’s *d* for stay probability on rare unrewarded trials = 0.42) between exercise and the task. The PTSD group also exhibited greater model-based behavior following the light-intensity short-delay condition compared to the light-intensity long-delay condition (Cohen’s *d* for stay probability on rare rewarded trials = −0.63; Cohen’s *d* for stay probability on rare unrewarded trials = 0.01). The control group exhibited greater model-based behavior following the moderate-intensity short-delay condition compared to the moderate-intensity long-delay condition (Cohen’s *d* for stay probability on rare rewarded trials = −0.34; Cohen’s *d* for stay probability on rare unrewarded trials = 0.13).

Analysis of simple effects indicated that the PTSD group exhibited greater model-based behavior when completing the task following moderate-intensity aerobic exercise compared to light-intensity aerobic exercise regardless of whether there was a short delay (*t*(7437) = 2.27, *p* = .023) or long delay (*t*(7188) = 5.79, *p* < .001) between exercise and the task (see Fig. 2A, Fig. 3D and Fig. 3E). Additionally, the PTSD group exhibited greater model-based behavior following the light-intensity short-delay condition compared to the light-intensity long-delay condition (*t*(7437) = −3.38, *p* < .001; see Fig. 2A and Fig. 3D), which is in contrast to the moderate-intensity session for which there was no difference in model-based behavior (although greater than light-intensity) between the short-delay and long-delay conditions (*t*(7188) = 1.16, *p* = .244; see Fig. 2A and Fig. 3E).

In contrast, the control group exhibited greater model-based behavior following the moderate-intensity short-delay condition compared to the moderate-intensity long-delay condition (*t*(7437) = −4.39, *p* < .001; see Fig. 2A and Fig. 3C), with no significant difference in model-based behavior following the light-intensity short-delay condition compared to the light-intensity long-delay condition (*t*(7437) = −1.60, *p* = .109; see Fig. 2A and Fig. 3B).

Finally, the control group exhibited greater model-based behavior during the light-intensity short-delay (*t*(7437) = −3.52, *p* < .001) and long-delay conditions (*t*(7437) = −4.46, *p* < .001) and the moderate-intensity short-delay condition (*t*(7437) = −3.16, *p* = .001) compared to the PTSD group, whereas the PTSD group exhibited greater model-based behavior during the moderate-intensity long-delay condition (*t*(7188) = 2.84, *p* = .004) compared to the control group (see Fig. 2B and Fig. 3B-E).

### 3.5 Supplemental analyses of possible confounding variables

Given that the PTSD group was on average older than the control group, we performed additional analyses to confirm that the aforementioned results associated with PTSD were not attributable to any group differences in age. We removed the minimal number of subjects from each group (3 youngest from control group and 3 oldest from PTSD groups) needed in order for there to no longer be a significant group difference in age before analyzing trial-by-trial stay vs switch behavior using identical logistic regression mixed models as described above. Results from these models with age-matched groups demonstrated similar effects as the primary analyses, as there was a significant reward x transition x experimental condition x delay condition x group interaction (*t*(23774) = 3.51, *p* < .001; see Supplemental Material and Fig. S4 for complete results). Additionally, site did not significantly interact with the primary effect of interest (i.e., significant reward x transition x experimental condition x delay condition x group interaction) and further statistical differences between sites were accordingly not explored (see Fig. S5 for figure depicting stay probabilities for each group [control and PTSD] across experimental manipulations [light-intensity vs moderate-intensity aerobic exercise and short delay vs long delay] separated by site [UW and UT]).

## 4 Discussion

The current study examined the extent to which moderate-intensity aerobic exercise promoted behavioral signatures of model-based learning in women with and without IPV-related PTSD, using a two-stage Markov decision-making task capable of distinguishing model-free and model-based behavioral strategies. The main findings from this study were that: 1) moderate-intensity aerobic exercise promotes greater model-based behavior during a two-stage Markov decision-making task compared to light-intensity aerobic exercise among women with PTSD, and 2) women without PTSD exhibit greater model-based behavior compared to women with IPV-related PTSD, although the gap in model-based behavior is reduced following moderate-intensity aerobic exercise (especially if the task is performed 30-minutes after the exercise bout has ended).

The current study provided the first evidence of greater engagement in model-based behavior during a two-stage Markov decision-making task following an acute bout of moderate-intensity aerobic exercise among women with PTSD. Importantly, the timing of when individuals completed the task in relation to exercise did not impair the effect of moderate-intensity aerobic exercise on model-based behavior among women with PTSD. In other words, moderate-intensity aerobic exercise promoted significantly greater model-based behavior compared to light-intensity aerobic exercise, regardless of whether the task was administered after a short delay or a longer delay of 30-minutes. The inclusion of a short-delay vs long-delay manipulation also revealed differential findings that varied as a function of PTSD diagnosis and our experimental manipulation (i.e., exercise intensity). For instance, the participants without PTSD that completed the task shortly after engaging in moderate-intensity aerobic exercise exhibited greater model-based behavior compared to those that completed the task after a longer delay of 30-minutes – an effect that was in the same direction (albeit non-significant) for light-intensity exercise. In contrast, the participants with PTSD that completed the task shortly after engaging in light-intensity aerobic exercise exhibited greater model-based behavior compared to those that completed the task after a longer delay, which was not the case for moderate-intensity exercise (i.e., no difference between delay conditions). Although these are between group differences (i.e., participants that completed exercise conditions after a short delay are a different set of participants that completed exercise conditions after a long delay), our data suggests that there may be a cap to the extent to which moderate-intensity aerobic exercise promotes model-based behavior in women with PTSD. Alternatively, it may be more appropriate to suggest that a delay of 30-minutes impairs the effects of moderate-intensity aerobic exercise on model-based behavior in women without PTSD and light-intensity in women with PTSD. Additionally, these results suggest that although moderate-intensity aerobic exercise is generally better for increasing model-based behavior, light-intensity aerobic exercise can exert an influence on model-based behavior among women with PTSD if the task is administered shortly after the light-intensity aerobic exercise bout has ended. This effect is especially visible in Fig. 3D, as those completing light-intensity aerobic exercise after a long delay exhibited the epitome of model-free behavior (as depicted in Fig. 3A).

Overall, the aforementioned findings are generally consistent with prior meta-analytic evidence [13] that suggests that: 1) cognitive tests administered shortly after an acute bout of aerobic exercise generally results in a greater effect on cognitive performance compared to when the cognitive task is performed following a longer delay (e.g., 20 min or more), and 2) when the cognitive task is performed following a delay of more than 1 min (which was the case for our study), more intense exercise intensities (moderate to vigorous) result in greater effects compared to lighter intensities (particularly those below 50% MHR). The exception to the aforementioned statement is that the control group exhibited similar model-based performance between light-intensity and moderate-intensity aerobic exercise - likely due to a ceiling effect on the maximum degree of model-based behavior possible. Relatedly, it should be noted that prior investigations into the effect of aerobic exercise on acute cognitive effects (i.e., those included in aforementioned meta-analysis) have not examined the effect of aerobic exercise on model-based learning [13], [48].

Although prior investigations had not directly tested the effect of aerobic exercise on promoting model-based learning – our findings are intuitive and consistent with several converging pieces of evidence. For instance, it is well-established that an acute bout of moderate-intensity aerobic exercise enhances performance on a number of cognitive tests, including executive function tasks [13], [14], [48]. This dovetails nicely with insight from functional brain imaging studies that have demonstrated that model-based learning engages the frontoparietal ‘central executive system’ that mediates executive function. In fact, prior research using transcranial magnetic stimulation (TMS) to enhance or diminish activity in the frontoparietal network has been found to enhance or diminish engagement of model-based learning [24], [25]. Relatedly, administration of the dopamine precursor (L-DOPA) has been shown to increase engagement of model-based learning compared to placebo [49], and exercise is also widely documented to boost dopamine signaling (in addition to targeting other neurotransmitters, neurotransmitter modulators, and peptides, such as neuropeptide-Y and endocannabinoids) [16], [19], [50], [51], [52], [53]. Future research is needed to examine the potential mediating and/or moderating role of several candidate biomarkers and neuromodulatory systems (e.g., neuropeptide-Y, endocannabinoids, dopamine) on the relationship between exercise and model-based behavior, which may also reveal important individual differences that inform precision medicine approaches (e.g., exercise may be most beneficial for enhancing model-based behaviors among individuals with greatest exercise-induced engagement in specific systems).

This study also provided initial support for greater model-based behavior in general among the control group compared to the PTSD group (with the exception of the moderate-intensity aerobic exercise condition performed after a delay). Model-based behavior requires the formation of cognitive representations of the environment in order to assist in goal-directed choices – a process that involves higher-level executive functioning (e.g., working memory, cognitive control, use of abstract rules or instruction) [4], [5], [6]. Given that individuals with PTSD often exhibit deficits in executive functioning compared to those without PTSD [7], [8], [9], [10], our finding of greater model-based behavior among the women without PTSD is consistent with prior research. Similarly, prior research has demonstrated that model-based behavior gradually increases as a function of developmental trajectory, such that it is absent in children, apparent in adolescents, and strengthened in adults [6]. This developmental progression in model-based behavior follows with the gradual development of executive functioning [54]. Unsurprisingly, the current study also found evidence for greater model-based behavior during participant’s second visit compared to their first visit. This finding is consistent with principles of model-based learning, in that experience with the task is expected to improve performance (i.e., promote model-based behavior). In fact, we specifically adopted the ‘magic carpet’ task [47] instructions to maximize participant knowledge of the task and minimize influence of incorrect task understanding on decision making during the task. As previously demonstrated, the implicit task instructions, quiz, and tutorial for the ‘magic carpet’ task resulted in a greater degree of model-based behavior (i.e., greater beta coefficients for reward x transition) compared to prior investigations that implemented different two-stage Markov tasks or instructions [6], [47]. In fact, it is plausible that the control group may have experienced somewhat of a ceiling effect, rendering it difficult for moderate-intensity aerobic exercise to exert an additional influence on performance within that group. Given that the PTSD group had lower model-based behavior in general, and saw greater model-based behavior following moderate-intensity aerobic exercise, our findings suggest that moderate-intensity aerobic exercise may exert more of an effect on promoting model-based behavior specifically among those with greater room for improvement.

Although additional research is warranted, our findings may offer preliminary support for including aerobic exercise as part of a comprehensive treatment approach for PTSD. Despite room for improvement, cognitive processing therapy (CPT) is a commonly administered and evidence-based psychological treatment for PTSD [55]. CPT focuses on exposure to the trauma memory as well as cognitive restructuring techniques (e.g., correcting maladaptive thoughts) [56], [57]. Importantly, an underlying assumption of treatment success is that individuals must form a correct model of their environment [58]. As such, it would follow that interventions capable of promoting model-based learning may enhance the efficacy of CPT among individuals with PTSD. More specifically, greater engagement of model-based behavior during CPT may allow individuals to correct maladaptive thought patterns and provide them with more flexibility (outside the clinical setting) to make better informed prospective predictions about likely outcomes of their decisions, which may allow them to do a better job at differentiating safety from threat, while simultaneously reducing avoidance behaviors and symptomatology more easily. From a clinical translational perspective, our results may suggest that administration of moderate-intensity aerobic exercise prior to individual CPT sessions may boost an individual’s ability to engage in model-based learning during the subsequent therapy session, thereby contributing to a greater degree of learning during the session, which may ultimately enhance treatment outcomes. Importantly, these findings also contribute to a broader line of research that suggest that the timing of exercise administration in relation to a therapy session may be highly dependent on the type of therapy being administered. For instance, whereas recent research suggest that aerobic exercise may be most effective for prolonged exposure therapy (another commonly administered evidence-based treatment for PTSD) if administered after each session (due to its effects on enhanced consolidation of extinction learning/new safety learning) [17], [59], our results suggests that aerobic exercise prior to CPT may be more effective as it may prime an individual to form more informed models during the therapy sessions. Clearly more research is needed to continue advancing our understanding of the potential for incorporating exercise into PTSD treatment.

The current study is not withstanding limitations. For instance, the current study only tested women with and without PTSD. It is unclear whether these results would apply to men (with and without clinical diagnosis) and women with other clinical diagnoses that may contribute to impaired baseline model-based behavior. Additionally, our sample included individuals who tended to have higher IQs. However, all of the aforementioned analyses in this manuscript controlled for IQ. Relatedly, we did not administer a more general assessment of executive functioning (or other neuropsychological constructs such as attentional control or impulse inhibition), which would have allowed us to examine whether exercise influenced general executive functioning, or if the effect of exercise was more specific to model-based behavior. Additionally, it is also possible that our results were confounded by participant’s executive functioning. It is imperative that future research assess executive functioning to address these limitations. Another limitation, although not unique to this study, is that it is necessary to have participants complete a large number of trials (and on two occasions) in order to get an accurate estimate of their behavior. Fortunately, we only had to exclude two participant’s data due to erroneous responding during the task, which arguably suggests that the rest of the participants remained relatively engaged in the task. It may be beneficial for future research to record participant’s behavior during the task, which would allow for analysis of participant engagement (e.g., eye tracking). Another limitation is that the PTSD group was significantly older than the control group. However, age was included as a fully interactive covariate in our analyses and we conducted supplementary analyses (with age-matched sample) to confirm that the results associated with PTSD were not attributable to any group differences in age. We also attempted to build in a generalization test by conducting the study at two sites. Nonetheless, additional replication of these results across different populations (e.g., different types of trauma, differing socioeconomic status, etc.) is necessary to further establish robustness of the observed effects. Finally, the current study solely focused on how aerobic exercise and PTSD impacts decision making in pursuit of reward. However, real world decision making (especially among individuals with PTSD) often involves concurrent and sometimes conflicting threat and reward outcomes. In other words, individuals may often have to determine whether pursuing a reward is beneficial even if there is a possibility that they may encounter a threat during the process of reward obtainment [12]. To further enhance the ecological validity of this task, future research should embed trials with threat outcomes (e.g., threatening stimuli, presence of shock) in order to determine if the presence of threat impacts the influence of aerobic exercise on model-based behavior in clinical and non-clinical populations. Additionally, one limitation with two stage Markov decision-making tasks is that they are unable to distinguish between model-free and *incorrect* model-based strategies. As such it is possible that participants with PTSD may have been forming incorrect mental models rather than exhibiting a greater degree of model-free behavior compared to the control group. However, even if this is the case, our results still suggest that the PTSD group exhibited greater *correct* model-based learning following moderate-intensity aerobic exercise compared to light-intensity aerobic exercise. Finally, despite the large number of repeated observations and a within-subjects manipulation, it is possible that our small sample size may have resulted in sample-specific non-generalizable findings. Future research with similar designs as the current study (i.e., between- and within-subjects manipulations in cases vs controls) should test a larger number of participants.

Overall, our results suggest that moderate-intensity aerobic exercise may be a promising manipulation for boosting model-based behavior in women with PTSD. Although further research is warranted, these results contribute to a broader line of research that suggest that aerobic exercise may play an important adjunctive role in enhancing the efficacy of commonly administered psychotherapies (e.g., prolonged exposure, cognitive processing therapy for PTSD and other anxiety disorders) due to its effect on enhancing relevant cognitive outcomes [16], [60], [61].

## Author contributions

All authors contributed meaningfully to the preparation of this manuscript. KMC led the conception and design of the work; contributed to the acquisition of the data; led the analysis and interpretation of the data for the manuscript; drafted and critically revised the work for important intellectual content; provided final approval of the version to be published; and is in agreement to be accountable for all aspects of the work. AA, CB, TSG, MH, JH, NM, and CS contributed to the acquisition of the data and provided critical revisions for important intellectual content. JMC led the conception and design of the work; provided substantial contributions to the analysis and interpretation of the data for manuscript; critically revised the work for important intellectual content; provided final approval of the version to be published; and is agreement to be accountable for all aspects of the work.

## Funding

This study was supported by the Virginia Horne Henry Fund at the University of Wisconsin – Madison awarded to KMC and JMC. KMC was supported by a National Institute on Alcohol Abuse and Alcoholism (NIAAA/NIH) training grant (T32AA007471) and is currently supported by the National Institute of Mental Health (K01MH132545). The funding sources had no role in the collection, analysis, and interpretation of data; in writing the report, or in the decision to submit the article for publication.

## Declaration of Competing Interest

The authors declare that they have no known competing financial interests or personal relationships that could have appeared to influence the work reported in this paper.

## Data Availability

The data that support the findings of this study are available from the corresponding author upon reasonable request.
