# Supplementary material for: The influence of aerobic exercise on model-based decision making in women with posttraumatic stress disorder
Source: J Mood Anxiety Disord. 2023 Jul 27;2:100015. doi: 10.1016/j.xjmad.2023.100015 (PMC10433398; doi:10.1016/j.xjmad.2023.100015)
Supplement: Supplementary file 1 — Supplementary material [file mmc1.pdf]

SUPPLEMENTARY MATERIAL

The influence of aerobic exercise on model-based decision making in women with posttraumatic stress disorder

Additional information regarding two-stage Markov task instructions and tutorial: During the instructions phase, participants were informed that they would be playing the role of a musician living in a fantasy land who played a musical instrument for genies (in an attempt to receive gold coins) who lived inside magic lamps on Pink and Blue Mountains. Two genies lived on each mountain and participants were told that the symbol (Tibetan character) written on each genie's lamp was the genie's name in the local language. In order to get to the mountains, participants were instructed to choose one of two magic carpets, which was labeled with two different symbols (Tibetan characters) that signified "Blue Mountain" and "Pink Mountain" in the local language. Participants were informed that a carpet would generally fly to the mountain whose name was written on it, but on rare occasions a strong wind blowing from the mountain would make flying there too dangerous, and as a result, the carpet would be forced to land on the other mountain. When the participants were on a mountain, they could select one of two lamps to pick up and rub. If the genie was interested in the musician's music, they would come out of their lamp and give the musician a gold coin. Participants were instructed that each genie's interest in music could change with time. Additionally, participants were instructed that the lamps on each mountain might switch sides between visits to a mountain because every time they picked up a lamp to rub it they might put it down in a different place. Similarly, participants were instructed that their carpets might switch sides from one trial to the next because each time they take them out of a cupboard, they might put them down in a different position.

Participants completed a practice quiz (seven multiple choice questions to verify that participants understood the task) and 25 practice trials (using different Tibetian symbols and orange and black lamps and mountains) during which they were told the meaning of each symbol on the carpet (i.e., explicitly knew which transition was common vs rare). This is in contrast to the actual task (which involved 250 trials), during which participants had to determine the meaning of each symbol on each carpet (i.e., had to figure out which carpet was more likely to go to each respective mountain), and were told that their carpets were upgraded to be entirely self-driving and that they would simply pick a carpet and then take a nap until they arrived at the mountain.

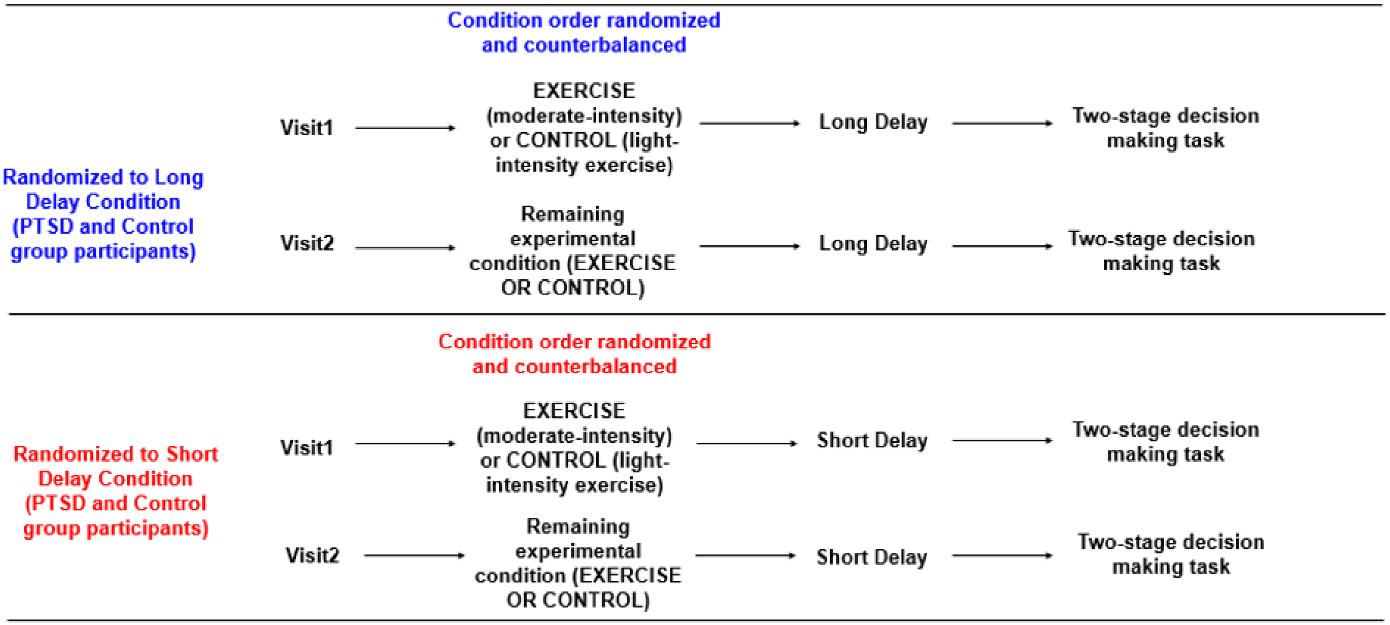

Figure S1. Detailed schematic of randomization procedures and experimental design. The current study involved within- and between-subjects randomization procedures. For the within-subject manipulation, participants were randomized to complete either light- or moderate-intensity aerobic exercise at study visit 1 (with remaining condition completed at study visit 2). For the between-subject manipulation, participants were randomized to complete the two-stage Markov decision making task either shortly after (short-delay condition; time to complete questionnaires and blood draw) or 30-minutes following (long-delay condition) following the exercise manipulation (i.e., following light- or moderate-intensity aerobic exercise). The randomization factors were crossed, resulting in 4 experimental groups. In other words, a quarter of participants completed the moderate-intensity/short-delay condition at study visit 1 (and the light-intensity/short-delay condition at study visit 2), a quarter of participants completed the moderate-intensity/long-delay condition at study visit 1 (and the light-intensity/long-delay condition at study visit 2), a quarter of participants completed the light-intensity/short-delay condition at study visit 1 (and the moderate-intensity/short-delay condition at study visit 2), and a quarter of participants completed the light-intensity/long-delay condition at study visit 1 (and the moderate-intensity/long-delay condition at study visit 2).

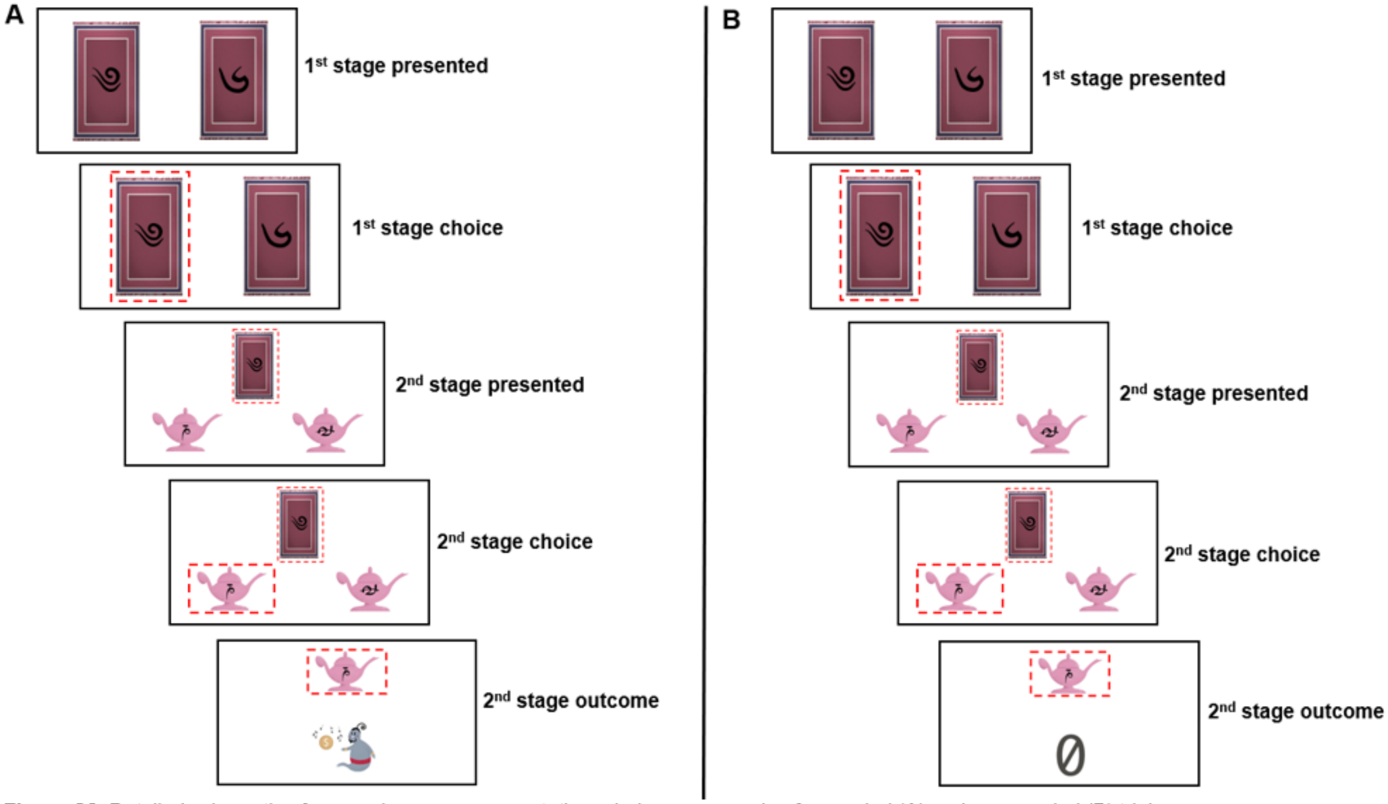

Figure S2. Detailed schematic of screen-by-screen presentations during an example of rewarded (A) and unrewarded (B) trials.

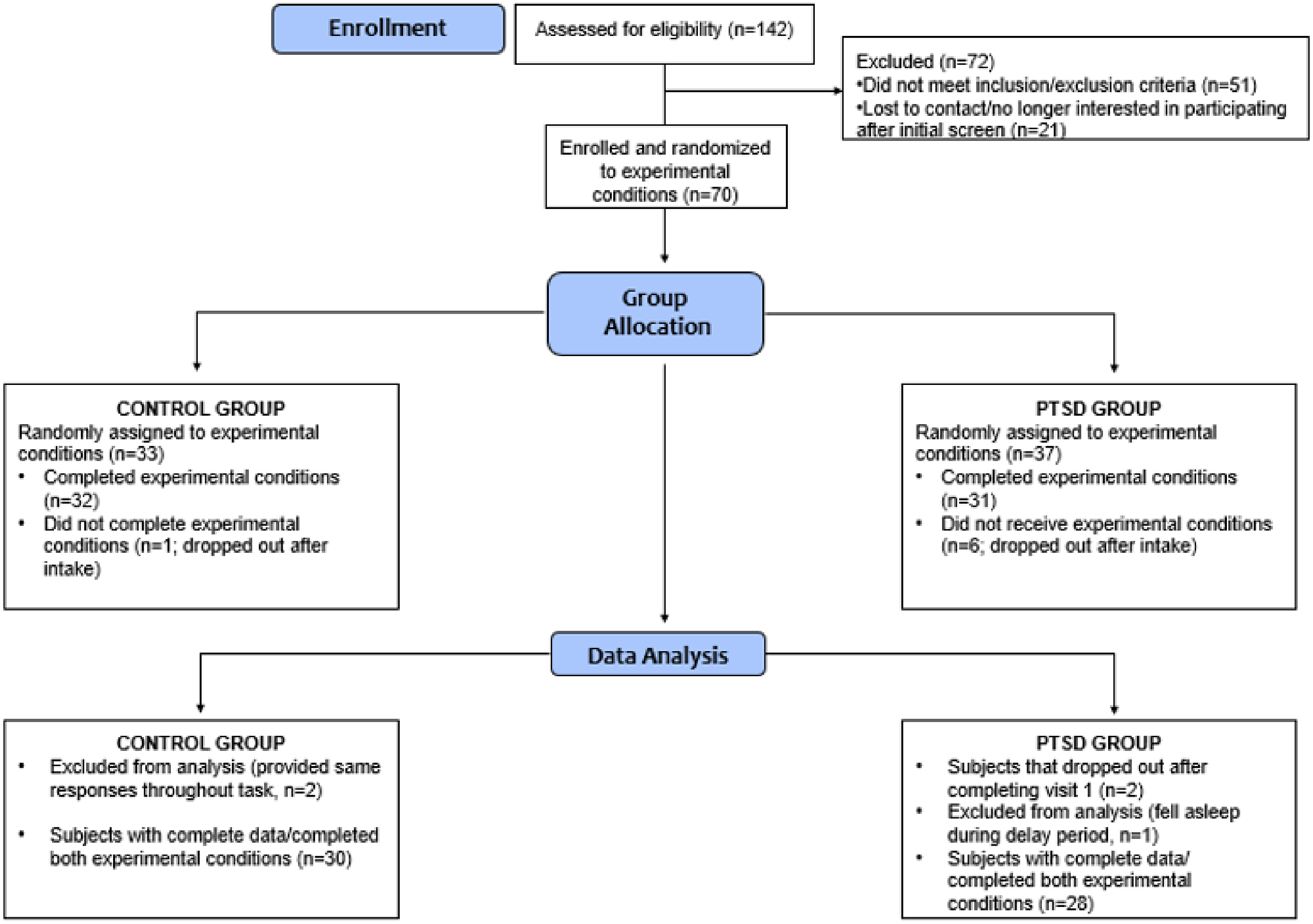

Figure S3. Consort diagram depicting enrollment of subjects, allocation to experimental groups, and data analyses procedures.

Participant characteristics: Additional secondary baseline variables characterizing the sample can be found in Table S1.

Table S1.
Group means and standard deviations for secondary baseline characteristics

	Control Group (n=30)				PTSD Group (n=31)				Between Group (CON vs PTSD) Statistics	
**Variable**	**Short Delay**	**Long Delay**	**t-statistic**	**p-value**	**Short Delay**	**Long Delay**	**t-statistic**	**p-value**	**t-statistic**	**p-value**
**Anxiety** (BAI)	7.47 ± 9.52	2.93 ± 2.37	1.79	0.084	14.33 ± 10.67	19.19 ± 8.75	-1.39	0.176	-5.25	<.001
**Sleep Quality** (PSQI)										
Subjective sleep quality	1.20 ± 0.68	0.80 ± 0.68	1.62	0.116	1.60 ± 0.74	1.81 ± 0.75	-0.80	0.433	-3.86	<.001
Sleep latency	1.27 ± 1.03	1.27 ± 1.67	0.00	0.999	2.00 ± 1.65	3.19 ± 2.07	-1.76	0.089	-3.12	0.003
Sleep duration	0.80 ± 0.77	1.00 ± 0.76	-0.72	0.480	1.47 ± 0.74	1.44 ± 0.96	0.09	0.926	-2.67	0.001
Habitual sleep efficiency	0.33 ± 0.72	0.53 ± 0.74	-0.75	0.462	0.87 ± 1.06	0.75 ± 1.00	0.32	0.755	-1.65	0.110
Sleep disturbances	1.13 ± 0.35	1.00 ± 0.38	1.00	0.326	1.73 ± 0.46	1.56 ± 0.51	0.98	0.337	-5.24	<.001
Use of sleeping medication	0.73 ± 1.28	0.40 ± 0.91	0.82	0.418	0.93 ± 1.10	0.81 ± 1.17	0.30	0.769	-1.07	0.289
Daytime dysfunction	0.87 ± 0.92	0.53 ± 0.52	1.23	0.230	1.67 ± 0.62	1.63 ± 0.50	0.21	0.837	-5.63	<.001
Global score	6.33 ± 4.10	5.53 ± 3.34	0.59	0.562	10.27 ± 3.63	11.19 ± 3.54	-0.71	0.481	-5.18	<.001
**Emotion Dysregulation** (DERS)										
Nonacceptance of emotional response	11.07 ± 5.40	10.80 ± 5.78	0.13	0.897	14.00 ± 7.02	15.00 ± 6.76	-0.40	0.689	-2.26	0.028
Difficulty engaging in goal-directed behavior	13.00 ± 4.69	12.00 ± 5.06	0.56	0.579	16.87 ± 4.81	18.38 ± 4.44	-0.91	0.371	-4.26	<.001
Impulse control difficulties	9.13 ± 3.07	7.87 ± 1.55	1.43	0.165	11.00 ± 3.82	14.69 ± 4.50	-2.45	0.024	-4.70	<.001
Lack of emotional awareness	13.93 ± 4.27	15.00 ± 5.69	-0.58	0.566	17.93 ± 4.56	17.31 ± 4.39	0.39	0.702	-2.62	0.011
Limited access to emotion regulation strategies	13.27 ± 3.88	11.06 ± 3.13	1.71	0.100	21.00 ± 5.80	21.00 ± 6.21	-1.05	0.303	-6.04	<.001
Lack of emotional clarity	10.47 ± 3.25	8.87 ± 2.39	1.54	0.135	11.80 ± 3.36	13.38 ± 2.94	-1.39	0.175	-3.75	<.001
Total	70.87 ± 17.81	65.60 ± 15.72	0.86	0.398	90.33 ± 19.98	99.75 ± 19.05	-1.34	0.19	-5.74	<.001
**Self-Reported PTSD symptom severity** (PCL-5)	-	-	-	-	33.53 ± 13.91	41.44 ± 13.55	-1.60	0.120	-	-

Note. BAI = Beck Anxiety Inventory; DERS = Difficulties in Emotion Regulation Scale (DERS); PCL-5 = PTSD Checklist for DSM-V.

Supplementary logistic regression analyses of stay/switch behavior with possible confounding variables: Given that the PTSD group was older than the control group, we performed additional analyses to confirm that the aforementioned results associated with PTSD were not attributable to any group differences in age. More specifically, we removed the minimal number of subjects from each group (3 youngest from control group and 3 oldest from PTSD groups) needed in order for there to no longer be a significant group difference in age ($t(47)=-1.19$, $p = .239$; control M ± SD = 31.63 ± 7.75, PTSD M ± SD = 34.28 ± 7.83) before analyzing trial-by-trial stay vs switch behavior using identical logistic regression mixed models as described in the main manuscript.

Similar to what was reported with the overall sample, there was a significant reward x transition x experimental condition x delay condition x group interaction ($t(23775)=3.86$, $p <.001$). As was done with the main analyses with the overall sample, we broke this interaction down by examining the simple effects separately within the PTSD and control groups, and then by examining differences between the PTSD and control groups. Analysis of simple effects indicated that the PTSD group exhibited greater model-based behavior when completing the task following moderate-intensity aerobic exercise compared to light-intensity

aerobic exercise regardless of whether there was a short delay (t(5943)=2.66, p =.007) or long delay (t(5943)=6.18, p < .001) between exercise and the task (see Figure S3 C). Additionally, the PTSD group exhibited greater model-based behavior following the light-intensity short-delay condition compared to the light-intensity long-delay condition (t(5943)=-4.48, p < .001; see Figure S3 C), which is in contrast to the moderate-intensity session for which there was no significant difference in model-based behavior (although greater than light-intensity) between the short-delay and long-delay conditions (t(5943)=0.93 p =.926; see Figure S3 C).

In contrast, the control group exhibited greater model-based behavior following the moderate-intensity short-delay condition compared to the moderate-intensity long-delay condition (t(5943)=-4.23, p < .001; see Figures S3 C), which is in contrast to the light-intensity session for which there was no significant difference in model-based behavior between short-delay and long-delay conditions (t(5943)=-1.17 p =.240; see Figure S3 C). Finally, the control group exhibited greater model-based behavior during the light-intensity short-delay (t(5943)=-2.39, p = .016) and long-delay conditions (t(5943)=-4.94, p < .001) compared to the PTSD group; whereas the PTSD group exhibited greater model-based behavior during the moderate-intensity long-delay condition (t(5943)=3.32, p < .001) compared to the control group (see Figure S3D). There was no significant difference between groups for the moderate-intensity short-delay condition (t(5958)=-1.32, p =.187).

Additionally, site did not significantly interact with the primary effect of interest (i.e., significant reward x transition x experimental condition x delay condition x group interaction) and further statistical differences between sites were accordingly not explored (see Figure S4 for figure depicting stay probabilities for each group [control and PTSD] across experimental manipulations [light-intensity vs moderate-intensity aerobic exercise and short delay vs long delay] separated by site [UW and UT]).

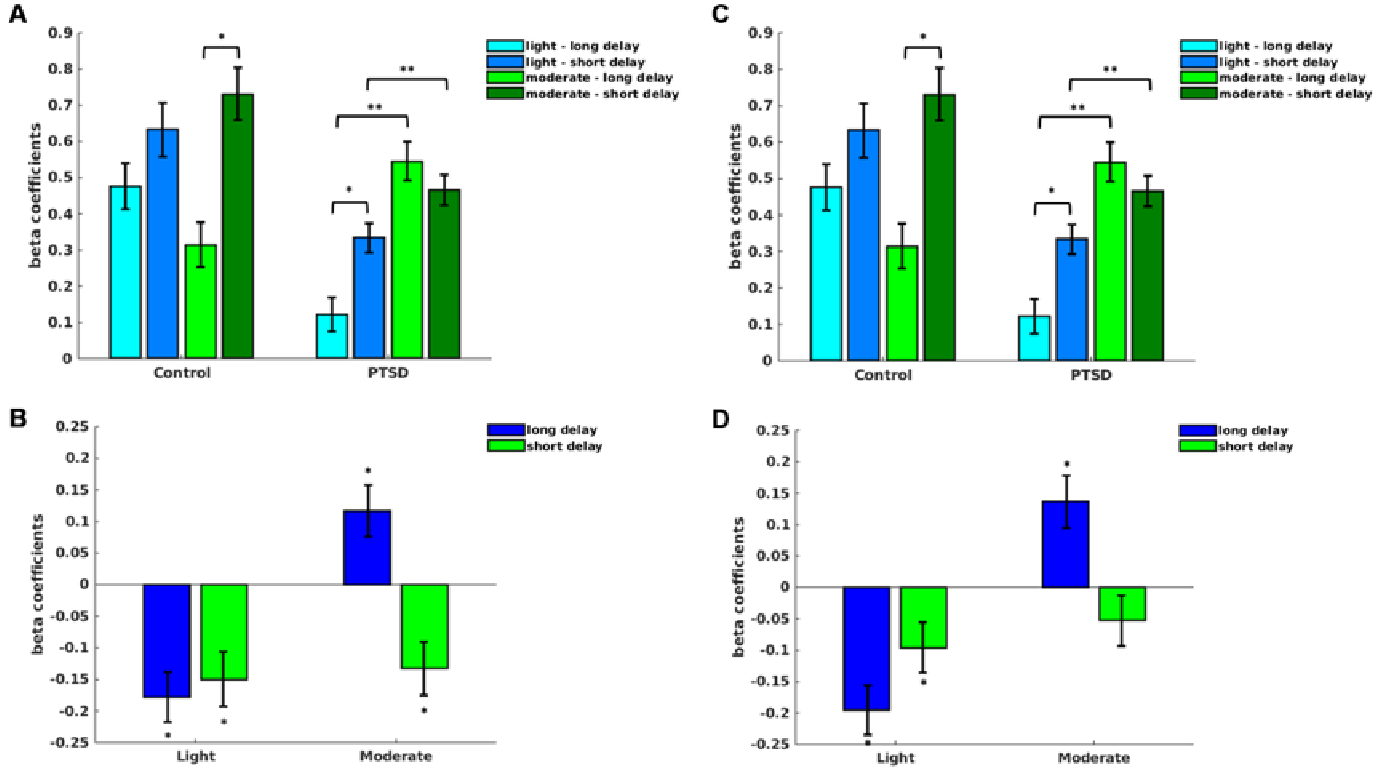

Figure S4. Figure depicting results from main and supplementary logistic regression analyses of stay/switch behavior during two-stage Markov decision-making task. Panel A and B depicts results from the overall sample (i.e., results reported in the main manuscript) and Panel C and D depicts supplementary results from age-matched groups (i.e., removed 3 youngest participants from control groups and 3 oldest participants from PTSD groups). Panel A beta-coefficients = reward x transition; Panel B beta-coefficients = reward x transition x group (control vs PTSD).

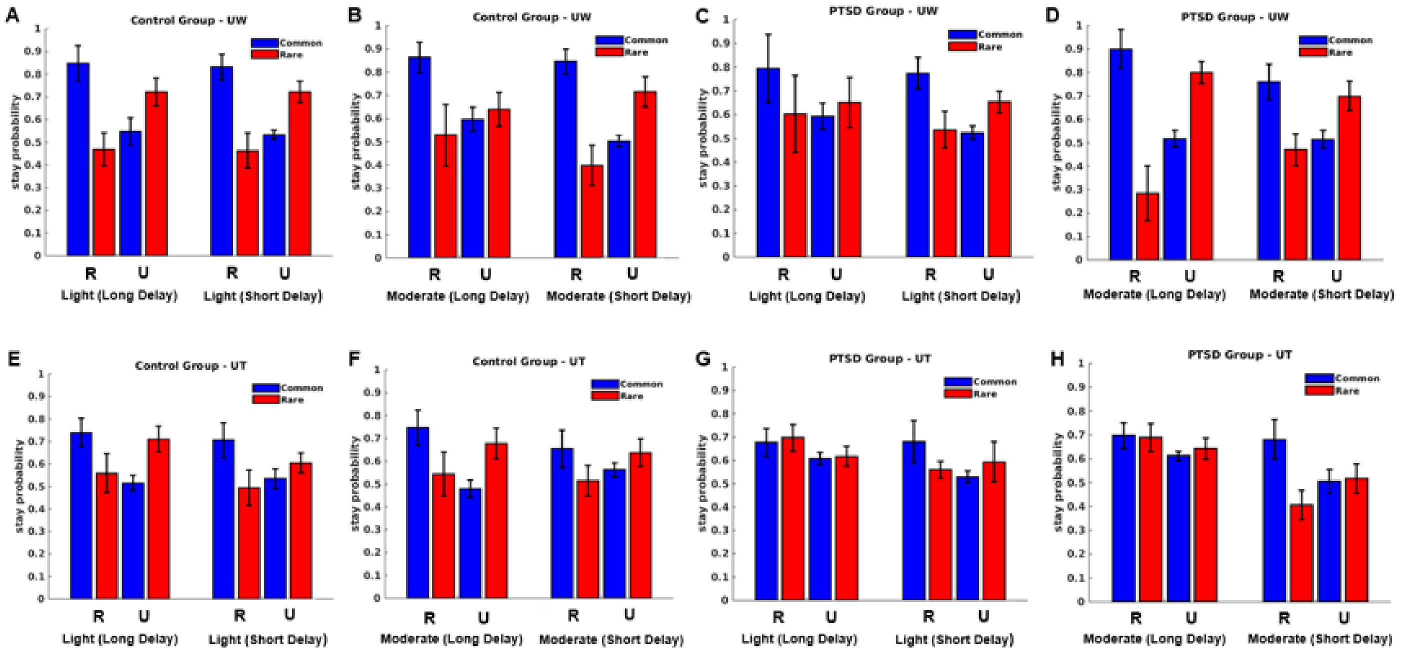

Figure S5. Figure depicting stay probabilities for each group (control and PTSD) across experimental manipulations (light-intensity vs moderate-intensity aerobic exercise and short delay vs long delay) separated by site (UW and UT). Panels A and B depicts stay probabilities for the UW control group participants during the light-intensity long delay and short delay conditions (see panel A) and during the moderate-intensity long delay and short delay conditions (see panel B). Panels C and D depicts stay probabilities for the UW PTSD group participants during the light-intensity long delay and short delay conditions (see panel C) and during the moderate-intensity long delay and short delay conditions (see panel D). Panels E and F depicts stay probabilities for the UT control group participants during the light-intensity long delay and short delay conditions (see panel E) and during the moderate-intensity long delay and short delay conditions (see panel F). Panels G and H depicts stay probabilities for the UT PTSD group participants during the light-intensity long delay and short delay conditions (see panel G) and during the moderate-intensity long delay and short delay conditions (see panel H). R = rewarded trials; U = unrewarded trials.

Description of Clinical Assessments and Baseline Questionnaires

Clinician Administered PTSD Scale (CAPS): The CAPS-5 [1] is a structured clinical interview developed by the National Center for PTSD and is considered the gold-standard structured clinical interview for assessing PTSD. The CAPS-5 contains 30 items that assess all of the diagnostic criteria for PTSD, including trauma exposure (criterion A), symptom severity (derived from symptom frequency and intensity) within each of the PTSD symptom clusters (Criterion B-E), and PTSD onset and duration, functional impairment, subjective distress, dissociative symptoms, and a clinician-estimated validity of the patient's responses provided during the assessment. The CAPS-5 was used to make a diagnosis of PTSD and assess PTSD symptom severity over the past month. The CAPS-5 symptom cluster severity scores were calculated by summing the individual item severity scores for symptoms corresponding to a given DSM-5 cluster: Criterion B (items 1-5); Criterion C (items 6-7); Criterion D (items 8-14); and, Criterion E (items 15-20).

National Women Survey Trauma Assessment (NSA): The NSA [2] assesses assaultive event exposure and other types of childhood adversity history and chronology.

Diagnostic Interview for Anxiety, Mood, and Compulsive Related Neuropsychiatric Disorders (DIAMOND): The DIAMOND [3] is a semi structured interview that targets the diagnostic criteria for a range of DSM-5 disorders, with additional clinical information gathered for the anxiety, mood, and obsessive- compulsive and related disorders.

Columbia-Suicide Severity Rating Scale (CSSRS): The CSSRS [4] quantifies the severity of suicidal ideation and behavior. The scale has been shown to exhibit good convergent and divergent validity with other suicidal ideation and behavior scales and exhibits high sensitivity and specificity for suicidal behavior classification compared with other scales and an independent suicide evaluation board [4].

Beck Depression Inventory-II (BDI-II): The BDI-II [5] is a 21-item self-report rating inventory that measures symptoms of depression. Participants responded to the 21-items using a 4-point Likert-type scale (0 = *not at all*, 1 = *mildly*, 2 = *moderately*, 3 = *severely*). Each item was summed to give a total depression score ranging from 0 to 63. A total score of 0-13 indicates minimal depression, 14-19 indicates mild depression, 20-28 indicates moderate depression, and 29-63 indicates severe depression. The BDI-II is one of the most widely used instruments for measuring depression and has been shown to be reliable and valid in both psychiatric and non-psychiatric populations [5,6].

Beck Anxiety Inventory (BAI): The BAI [7] is a validated 21-item self-report rating inventory used for measuring anxiety. Participants responded to the 21-items using a 4-point Likert-type scale (0 = not at all, 1 = mildly, 2 = moderately, 3 = severely). Like the BDI-II, each item was summed to give a total anxiety score ranging from 0 to 63. Cut scores have been established, with a summed score of 0-9 indicating minimal anxiety, 10-16 indicating mild anxiety, 17-29 indicating moderate anxiety, and 30-63 indicating severe anxiety. The BAI is one of the most widely used instruments for measuring anxiety and has been shown to be reliable and valid in both psychiatric and non-psychiatric populations.

Posttraumatic Stress Disorder Checklist-5 (PCL-5): The PCL-5 [8] is a self-report PTSD symptom questionnaire that was administered in order to assess PTSD symptom severity. The PCL-5 is a standardized self-report rating scale used in clinical and research settings for screening, diagnosing, and monitoring symptom change regarding PTSD. The PCL-5 is comprised of 20 items corresponding to the key symptoms of PTSD. Participants were asked to indicate how much they have been bothered by a symptom over the past month using a 5-point Likert scale (0 = not at all, 1 = a little bit, 2 = moderately, 3 = quite a bit, 4 = extremely). A total symptom severity score ranging from 0 – 80 was obtained by summing the scores from each of the 20 items. The PCL-C has been shown to be reliable and valid in civilians and military personnel and veterans [8–10].

Childhood Trauma Questionnaire (CTQ): The CTQ [11] measures histories of abuse and neglect during childhood.

Difficulty in Emotion Regulation Scale (DERS): The DERS [12] assesses multiple aspects of emotion dysregulation via 36 items rated on a 5-point Likert scale. The DERS has the following subscales, including: nonacceptance of emotional response, difficulty engaging in goal-directed behavior, impulse control difficulties, lack of emotional awareness, limited access to emotion regulation strategies, and lack of emotional clarity; in addition to a total score. The DERS has high internal consistency, good test–retest reliability, and adequate construct and predictive validity [12].

Pittsburgh Sleep Quality Index (PSQI): The PSQI [13] is an effective instrument to measure the quality and patterns of sleep in adults. The PSQI contains 19 questions measuring seven areas (components): subjective sleep quality, sleep latency, sleep duration, habitual sleep efficiency, sleep disturbances, use of sleeping

medications, and daytime dysfunction over the last month. The PSQI has been found to be reliable and valid in the assessment of self-reported sleep problems [14].

Mood and Anxiety Symptoms Questionnaire (MASQ D-30): THE MASQ [15] measures general (non-disorder specific) symptoms of mood dysfunction and anxiety.

Perceived Stress Scale (PSS): The PSS [16] is the most widely used reliable and valid psychological instrument for measuring the perception of stress. It is a measure of the degree to which situations in one's life are appraised as stressful. Items were designed to quantify how unpredictable, uncontrollable, and overloaded respondents find their lives over the past month. Higher PSS scores are indicative of greater perceived stress. The PSS has been applied in a variety of study populations. The PSS demonstrates high test-retest reliability (ICC = 0.85) and has been correlated with other similar measures (0.52-0.76).

State Trait Anxiety Inventory – State Version (STAI): The STAI [17] is a widely used self-report measure that indicates the experienced intensity of feelings of anxiety. The state version of the STAI contains 20 items (e.g., "I feel at ease") assessing present levels of anxiety. Participants responded to each item on a Likert-type scale (1 = *not at all,* 2 = *somewhat,* 3 = *moderately,* 4 = *very much so*). Each of the 20 items are summed to give a total state anxiety score (ranging from 20-80; adult population M = 35.2; SD = 10.6), with higher scores indicating greater anxiety. The STAI is valid and reliable with high internal consistencies ranging from 0.86-0.95.

Positive and Negative Affect Schedule (PANAS): The PANAS [18] is a validated 20-item questionnaire that comprises two mood scales: one measuring positive affect (10 items), and one measuring negative affect (10 items). Each of the 20 items were rated on a 5-point scale (1 = *very slightly or not at all*, 2 = *a little*, 3 = *moderately*, 4 = *quite a bit*, 5 = *extremely*) to indicate the extent to which the participant felt positive or negative affect during the indicated time frame. For the purpose of this study, participants were asked to respond to the 20-items based on how they feel "at this moment." Responses to the items were summed in order to obtain a total positive affect (adult population M = 31.31, SD = 7.65) and negative affect (adult population M = 16.00, SD = 5.90) score, each ranging from 10-40 [19].

References

1. Weathers FW, Blake DD, Schnurr PP, Kaloupek DG, Marx BP, Keane TM. The Clinician-Administered PTSD Scale for DSM-5 (CAPS-5). 2013.
2. Kilpatrick DG, Ruggiero KJ, Acierno R, Saunders BE, Resnick HS, Best CL. Violence and risk of PTSD, major depression, substance abuse/dependence, and comorbidity: results from the National Survey of Adolescents. J Consult Clin Psychol. 2003;71:692–700.
3. Tolin DF, Gilliam C, Wootton BM, Bowe W, Bragdon LB, Davis E, et al. Psychometric Properties of a Structured Diagnostic Interview for DSM-5 Anxiety, Mood, and Obsessive-Compulsive and Related Disorders. Assessment. 2018;25:3–13.
4. Posner K, Brown GK, Stanley B, Brent DA, Yershova KV, Oquendo MA, et al. The Columbia–Suicide Severity Rating Scale: Initial Validity and Internal Consistency Findings From Three Multisite Studies With Adolescents and Adults. Am J Psychiatry. 2011;168:1266–1277.
5. Beck AT, Steer RA, Brown GK. Beck Depression Inventory Manual (2nd ed.). San Antonio, TX: Psychological Corporation; 1996.
6. Beck AT, Steer RA. Internal consistencies of the original and revised Beck Depression Inventory. J Clin Psychol. 1984;40:1365–1367.
7. Beck AT, Steer RA. Beck Anxiety Inventory Manual. San Antonio, TX: Psychological Corporation; 1993.
8. Blevins CA, Weathers FW, Davis MT, Witte TK, Domino JL. The Posttraumatic Stress Disorder Checklist for DSM-5 (PCL-5): Development and Initial Psychometric Evaluation. J Trauma Stress. 2015;28:489–498.
9. Bovin MJ, Marx BP, Weathers FW, Gallagher MW, Rodriguez P, Schnurr PP, et al. Psychometric properties of the PTSD Checklist for Diagnostic and Statistical Manual of Mental Disorders-Fifth Edition (PCL-5) in veterans. Psychol Assess. 2016;28:1379–1391.
10. Wortmann JH, Jordan AH, Weathers FW, Resick PA, Dondanville KA, Hall-Clark B, et al. Psychometric analysis of the PTSD Checklist-5 (PCL-5) among treatment-seeking military service members. Psychol Assess. 2016;28:1392–1403.
11. Bernstein DP, Fink L, Handelsman L, Foote J, Lovejoy M, Wenzel K, et al. Initial reliability and validity of a new retrospective measure of child abuse and neglect. Am J Psychiatry. 1994;151:1132–1136.
12. Gratz KL, Roemer L. Multidimensional Assessment of Emotion Regulation and Dysregulation: Development, Factor Structure, and Initial Validation of the Difficulties in Emotion Regulation Scale. Journal of Psychopathology and Behavioral Assessment. 2004;26:41–54.
13. Buysse DJ, Reynolds CF, Monk TH, Berman SR, Kupfer DJ. The Pittsburgh Sleep Quality Index: a new instrument for psychiatric practice and research. Psychiatry Res. 1989;28:193–213.
14. Mollayeva T, Thurairajah P, Burton K, Mollayeva S, Shapiro CM, Colantonio A. The Pittsburgh sleep quality index as a screening tool for sleep dysfunction in clinical and non-clinical samples: A systematic review and meta-analysis. Sleep Med Rev. 2016;25:52–73.
15. Clark LA, Watson D. Tripartite model of anxiety and depression: psychometric evidence and taxonomic implications. J Abnorm Psychol. 1991;100:316–336.
16. Cohen S, Kamarck T, Mermelstein R. A global measure of perceived stress. J Health Soc Behav. 1983;24:385–396.
17. Spielberger CD, Gorsuch RL, Lushene R, Vagg PR, Jacobs GA. Manual for the State-Trait Anxiety Inventory. Palo Alto, CA: Consulting Psychological Press; 1983.
18. Watson D, Clark LA, Tellegen A. Development and validation of brief measures of positive and negative affect: the PANAS scales. J Pers Soc Psychol. 1988;54:1063–1070.
19. Crawford JR, Henry JD. The positive and negative affect schedule (PANAS): construct validity, measurement properties and normative data in a large non-clinical sample. Br J Clin Psychol. 2004;43:245–265.
